# Sleep onset time as a mediator in the association between screen exposure and aging: a cross-sectional study

**DOI:** 10.1007/s11357-024-01321-x

**Published:** 2024-08-27

**Authors:** Senlin Lin, Meng Gao, Juzhao Zhang, Yuting Wu, Tao Yu, Yajun Peng, Yingnan Jia, Haidong Zou, Lina Lu, Deshang Li, Yingyan Ma

**Affiliations:** 1 Shanghai Eye Diseases Prevention &Treatment Center/ Shanghai Eye Hospital, School of Medicine, Tongji University, No. 1440, Hongqiao Road, Shanghai, 200336 China; 2 National Clinical Research Center for Eye Diseases, Shanghai, China; 3 Shanghai Engineering Research Center of Precise Diagnosis and Treatment of Eye Diseases, Shanghai, China; 4 Sijing Community Health Service Center, Shanghai, China; 5 School of Public Health, Shanghai Jiao Tong University School of Medicine, Shanghai, 200025 China; 6 Key Lab of Public Health Safety of the Ministry of Education, School of Public Health, Fudan University, 130 Dongan Road, Shanghai, 200032 China; 7 Health Communication Institute, Fudan University, Shanghai, 200032 China; 8 Department of Ophthalmology, Shanghai General Hospital, Shanghai Jiao Tong University School of Medicine, No. 100, Haining Road, Shanghai, 200080 China; 9 Shihudang Community Health Service Center, No. 1 to 5, Lane 50, Yanshou Road, Shanghai, China

**Keywords:** Screen exposure, Aging, Sleep, Retinal age

## Abstract

Excessive screen exposure has become a significant health concern. This study investigates the impact of screen time on aging in middle-aged and elderly populations. Healthy working adults over 45 years old in Shanghai, China, underwent general and ocular examinations. Questionnaires collected demographics, medical history, and screen exposure details. Aging was assessed using the retinal age gap, defined as the difference between the retinal age predicted by deep learning algorithms based on fundus images and chronological age. Pathway analysis tested the mediation effect of sleep duration and onset time on the relationship between screen usage and retinal age gap. The retinal age gap increased with longer screen exposure, from 0.49 ± 3.51 years in the lowest tertile to 5.13 ± 4.96 years in the highest tertile (Jonckheere-Terpstra test, *p* < 0.001). Each additional hour of screen exposure accelerated the retinal age gap by 0.087 years (95% CI, 0.027, 0.148, *p* = 0.005) in the fully adjusted linear model. Sleep onset time mediated the impact of screen usage on the retinal age gap (indirect effect, *β* = 0.11; 95% CI 0.04–0.24). The impact of screen usage in a light-off environment on the retinal age gap was fully mediated by sleep onset time (indirect effect, *β* = 0.22; 95% CI 0.07–0.38), with the proportion being 100%. Our study identified a correlation between excessive screen time and a wider retinal age gap in middle-aged and elderly individuals, likely due to delayed sleep onset. To mitigate the adverse effects on the retina and aging, it is important to limit screen usage and avoid screens before bedtime.

**Supplementary Information:**

The online version contains supplementary material available at 10.1007/s11357-024-01321-x.

## Background

Excessive exposure to screen devices, including smart phones, computer, and television, which are frequently equipped with light-emitting diodes (LED), becomes a pervasive global health concern in recent decades. Mounting evidence indicates that prolonged screen time was associated with ocular diseases, such as myopia, dry eye, and digital eye strain [1–3]. Additionally, extended screen time was also reported to have negative impact on other health problems, such as increased risk of obesity, mental health, disrupted circadian rhythm, cardiovascular diseases, and stroke [4–8]. It is now well recognized the adverse effect of excessive screen time on both physiological and psychological health in children and adolescents [5, 9]; however, studies on the effect of screen time and health problems in middle-aged and elderly population are limited [8].

Among the various health effects of screen exposure, it is worth noting its impact on sleep and circadian rhythms [6, 10]. The luminescence spectrum of screen devices is mainly concentrated in the short-wavelength blue light band around 450 nm, which is generally more effective than long-wavelength light in inhibiting melatonin levels, altering circadian rhythms, increasing alertness, and decreasing subsequent sleep quality [11]. The existing literature suggested that long-time exposure to screen can lead to shorter sleep duration, delayed sleep onset time, deteriorated sleep quality, and other manifestations of circadian rhythm disorder [6, 10, 12, 13]. Sleep and aging are closely related [14]. Normal aging is accompanied by a reduced capacity to initiate and maintain sleep [14]. Circadian and sleep dysfunction could be early signs of various neurodegenerative diseases, and be involved in driving the process of brain aging [15, 16]. Moreover, sleep and circadian disorders are also associated with accelerated epigenetic aging, shorter telomere length, and other hallmarks of biological aging [17–19].

The fundus is the only part of the human body where nerves and blood vessels are visible without invasive methods [20]. The morphological changes in retinal structures such as the optic disc and blood vessels not only represent the occurrence and development of ocular diseases, but also are indicators for general health. For example, changes in the diameter of retinal vessels are considered to be associated with systemic diseases such as hypertension, arteriosclerosis [21], chronic kidney diseases [22], and stroke. Recently, retinal age, developed using deep learn technology based the fundus images, has been verified as a novel biomarker for aging [23]. The retinal age gap was calculated as the predicted age derived from the fundus image minus the chronological age, which has been shown to be associated with the risk of mortality [23], Parkinson’s disease [24], cardiovascular diseases [25, 26], metabolic syndrome [27], diabetic retinopathy, and kidney failure [28, 29]. To the best of our knowledge, there is no study reporting the association between screen exposure and retinal age gap.

Therefore, we speculate that excessive screen exposure may lead to accelerated aging by disrupting circadian rhythm such as shorter sleep duration and delayed sleep onset. We intend to verify this hypothesis through investigating the relationship between screen time, sleep duration and onset and retinal age gap in a middle-aged and elderly population.

## Methods

### Study design and population

This is a cross-sectional study in 2023 in Shanghai, China. We randomly selected one community in Shanghai Songjiang District. We recruit healthy working people over the age of 45 years. Exclusion criteria were as follows: (1) those with severe systemic diseases, such as tumor, autoimmune diseases, history of myocardial or cerebral infarction, kidney or hepatic dysfunction; (2) those with severe ocular diseases, such as glaucoma, age-related macular degeneration, pathologic myopia, and grade 3 or higher cataract based on Emery classification; (3) those cannot cooperate with the examinations or questionnaires; and (4) those with incomplete examination results; (5) those who work outdoors or in strong light.

### General and ocular examinations

All participants underwent general examinations and ocular examinations. General examinations included height, weight, hip circumference, heart rate, blood pressure, and fasting blood tests. Blood tests contained fasting blood glucose, triglyceride, total cholesterol, high-density lipoprotein, low-density lipoprotein, and serum creatinine. Blood pressure was defined as the mean of two repeated measures with at least a 1-min interval taken with a HEM-7015IT electronic blood pressure monitor (Omron Healthcare, the Netherlands) in a seated position.

Ocular examinations included visual acuity, refraction, and fundus photography. The fundus photography was carried out using standardized methods and a unified parameter setting. For each participant, after 5 min of dark adaptation, the optic disc and macula of both eyes were photographed in a darkened room using a 50° 3.78-megapixel digital nonmydriatic camera (Kestrel-3100 m, SYSEYE, Chongqing, China). Since the correlation between the right and the left eye was high [30], to ensure the independency of the observation, optic disc-centered photos of the right eye were used for analyses, and if lacking or not qualified, the photos from the left eye were used instead.

### Questionnaires and measurement of screen exposure time

Variables on demographics, lifestyle, and medical history were obtained through questionnaires. Basic information such as birth date, gender, job, education level, and marriage status were collected. Medical history, including hypertension, hyperlipidemia, diabetes, and other systemic and ocular diseases were also collected. Average outdoor activity duration and usual sleep time in the most recent month were recorded. We requested the participants to provide precise timings (at what time and minutes, e.g., 10:30 p.m.) of their usual sleep onset and wake-up times during the day and at night for the past 1 month.

Additionally, detailed information about the type of screen exposure and the corresponding duration in the light-on or the light-off environment in the most recent month was recorded. The type of screen exposure included mobile phone, computer, television, projector, and others. Participants were required to specify the exact duration of time spent using each type of electronic device, separately for usage in the light-on environment and in the light-off environment. For instance, regarding mobile phone usage, participants were asked to record the length of time spent using the phone in light-on and light-off environment, respectively. The participants were also asked to choose the content of the screen exposure, such as social communication, reading books, watching short videos, watching movies or TV series, and playing games.

### Fundus images, deep learning model for age prediction, and calculation of retinal age gap

We formerly had developed a deep learning (DL) model based on color fundus images for age prediction (Supplementary material 1). Upon completion of the model training, predictions were made on an additional 664 images, and the retinal age gap was calculated. The retinal age gap is defined as the difference between the retinal age predicted by the DL model and the actual age. A positive retinal age gap indicates a retina that may be aging faster than normal, while a negative retinal age gap suggests the opposite. The average age of the population from which this set of images was sourced is 55.96, with a standard deviation of 4.72, a maximum age of 62, and a minimum age of 45.

### Statistical analysis

The baseline features were summarized through descriptive statistics. Continuous variables were expressed as mean ± standard deviation or median (interquartile range), while categorical variables were presented as counts (percentages). Weight (in kilograms) divided by squared height (in meters) was used to calculate BMI. Mean arterial pressure was calculated as (systolic pressure + 2×diastolic pressure)/3. Total screen exposure time was the sum of all the screen usage types and their corresponding duration. The total duration of screen exposure time was also separately calculated for both light-on and light-off environments. The variable “sleep onset time” was designated to represent the time of sleep onset at night. Using 0:00 as a starting point, we calculated how many hours have passed since the time of sleep onset from 0:00. For example, if sleep onset at 10:30 p.m., we would record it as 22.5 h; if sleep onset at 0:15 a.m. of the next day, we would record it as 24.25 h. The interval between sleep onset time and wake-up time, including both during the day and at night, is designated as the variable “sleep duration.”

The continuous variables were in the form of mean (standard deviation) and were statistically tested by one-way ANOVA for comparing three groups and by Student’s *t*-test for comparing two groups; the categorical variables were in the form of No. (%), and were analyzed using chi-squared test. To test the trend of the three groups, Jonckheere-Terpstra test was used for continuous variables and Cochran-Mantel–Haenszel test was used for ordinal variables. Multiple linear regression was used for assessing the associations of screen usage duration with retinal age gap. Model 1 was adjusted for age, gender, and education. Model 2 was further adjusted for time outdoors, sleep duration, sleep onset time, fasting blood glucose, BMI, hip circumference, total cholesterol, and mean arterial pressure. Pathway analysis was used to test the mediation effect of sleep duration (or sleep onset time) between screen usage and retinal age gap. Bootstrap confidence intervals were performed [31]. Subgroup analysis was performed by age group. SAS version 9.4 (SAS Institute, Cary, NC) was used to conduct all the analyses. Two-sided *P*-values of 0.05 were deemed significant if not otherwise indicated.

## Results

### Basic characteristics of the study population

**Fig. 1:**
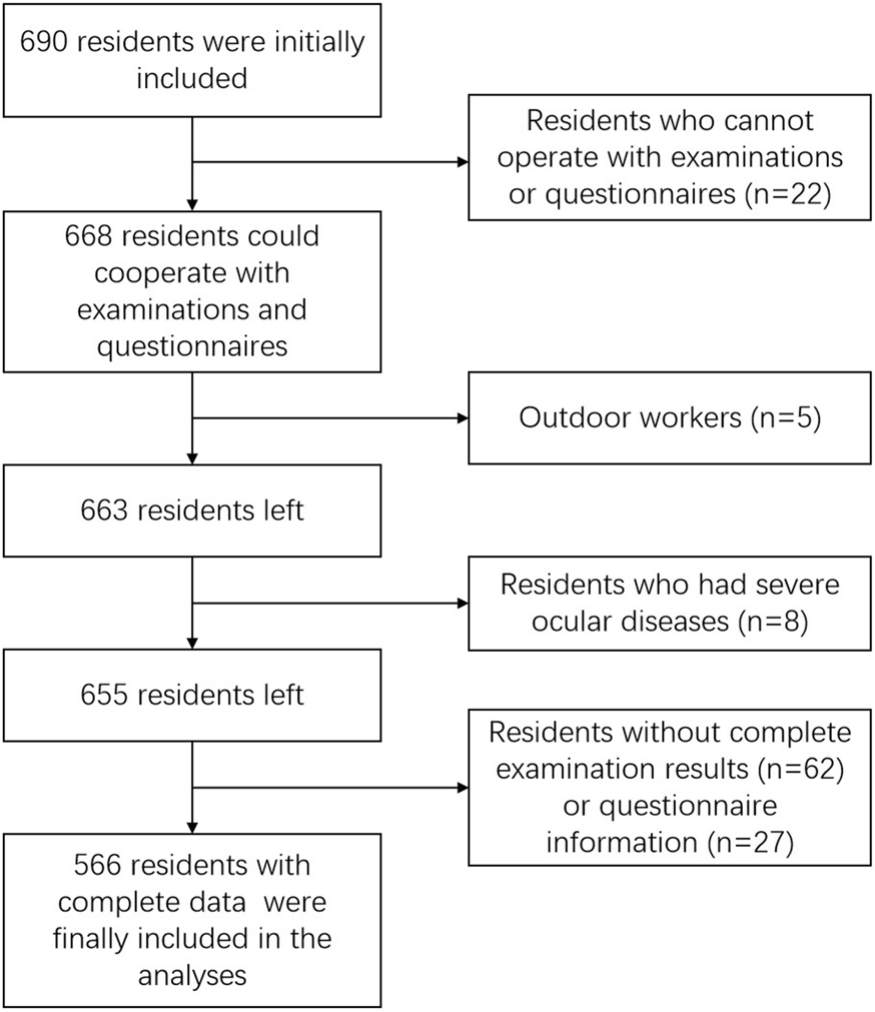
Enrollment flowchart of the participants

A total of 690 residents were initially recruited, among which 5 residents who work outdoors under intense light, 8 residents with severe eye diseases, 22 residents who could not cooperate with the examinations or questionnaire surveys, and 89 residents without complete examination results or questionnaire information were excluded (Fig. 1). As a result, 566 residents were included, comprising 194 males (34.28%) and 372 females (65.72%), with an overall mean age of 55.42 ± 4.78 years. The average total daily screen exposure time was 4.55 ± 2.16 h, with 4.46 ± 2.11 h in the light-on environment and 0.09 ± 0.3 h in the light-off environment.

Table 1 shows the basic characteristics of the study population stratified by the tertiles of the total screen exposure time. Retinal age gap, chronological age, gender, education level, sleep duration, sleep onset time, and outdoor time were statistically different among the three groups. The groups with higher screen exposure time were younger (ANOVA, *p* < 0.001; Jonckheere-Terpstra, *p* < 0.001), received higher educational level (*p* < 0.001; Cochran-Mantel–Haenszel, *p* < 0.001), shorter outdoor time (ANOVA, *p* < 0.001; Jonckheere-Terpstra, *p* < 0.001), shorter total sleep duration (ANOVA, *p* = 0.040; Jonckheere-Terpstra, *p* = 0.008), especially for the night-time sleep (ANOVA, *p* = 0.005; Jonckheere-Terpstra, *p* < 0.001), and later sleep onset (ANOVA, *p* < 0.001; Jonckheere-Terpstra, *p* < 0.001).

**Table 1:** Basic characteristics stratified by tertiles of total screen exposure time ^a^

Variables	Screen exposure time			Total (*n* = 566)	*P*-value
	Low (*N* = 184)	Median (*N* = 211)	High (*N* = 171)		
Screen exposure time (hours)					
Total	2.28 (0.79)	4.47 (0.49)	7.07 (1.6)	4.55 (2.16)	< 0.001
Light-on	2.27 (0.8)	4.38 (0.53)	6.92 (1.59)	4.46 (2.11)	< 0.001
Light-off	0.01 (0.11)	0.1 (0.29)	0.16 (0.4)	0.09 (0.3)	< 0.001
Retinal age gap (years)	0.49 (3.51)	2.79 (4.56)	5.13 (4.96)	2.75 (4.75)	< 0.001
Chronological age (years)	57.53 (3.46)	55.48 (4.63)	53.07 (5.12)	55.42 (4.78)	< 0.001
Outdoor duration (hours)	2.16 (1.38)	1.5 (1.13)	1.12 (0.93)	1.6 (1.24)	< 0.001
Sleep duration (hours)					
Total	7.86 (0.55)	7.87 (0.62)	7.72 (0.72)	7.82 (0.63)	0.040
Day	0.07 (0.22)	0.12 (0.35)	0.12 (0.34)	0.1 (0.31)	0.267
Night	7.79 (0.52)	7.75 (0.57)	7.6 (0.6)	7.72 (0.57)	0.005
Glucose (mmol/L)	6.01 (1.23)	6.08 (1.51)	5.99 (1.89)	6.03 (1.55)	0.845
BMI (kg/m^2^)	24.57 (3.1)	24.49 (2.95)	24.29 (3.49)	24.46 (3.17)	0.702
Hip circumference (cm)	82.25 (8.89)	81.17 (8.66)	81.35 (9.65)	81.57 (9.04)	0.459
Total cholesterol (mmol/L)	5.28 (1.02)	5.43 (1.04)	5.27 (0.93)	5.33 (1)	0.210
Mean arterial pressure (mmHg)	100.55 (10.03)	99.45 (11.27)	99.88 (12.46)	99.94 (11.26)	0.624
Sleep onset (time)	22.16 (0.62)	22.49 (0.62)	22.79 (0.62)	22.48 (0.67)	< 0.001
Gender No. (%)					0.024
Male	74 (40.22)	58 (27.49)	62 (36.26)	194 (34.28)	
Female	110 (59.78)	153 (72.51)	109 (63.74)	372 (65.72)	
Education No. (%)					< 0.001
Middle school and below	138 (75)	108 (51.18)	53 (30.99)	299 (52.83)	
High school and above	46 (25)	103 (48.82)	118 (69.01)	267 (47.17)	

^a^The basic characteristics of the study population were presented according to the tertiles of the screen exposure time. The participants’ screen exposure time was below 4 h in the low group, between 4 and 5 h in the median group, and more than 5 h in the high group. Since some residents shared the same amount of screen exposure time, the sample size of the three groups was not equally distributed. The continuous variables were in the form of mean (standard deviation) and were statistically tested by one-way ANOVA; the categorical variables were in the form of No. (%), and were analyzed using chi-squared test. *BMI* body mass index

Table 2 shows the basic characteristics of the study population stratified by median of retinal age gap. Screen exposure time, chronological age, education level, outdoor time, sleep duration, sleep onset time, and hip circumference were statistically different among the two groups. The group with larger retinal age gap was younger (*p* < 0.001), received higher educational level (*p* < 0.001), shorter outdoor time (*p* < 0.001), shorter total sleep duration (*p* < 0.001), especially for the night-time sleep (*p* < 0.001), later sleep onset (*p* < 0.001), and smaller hip circumference (*p* = 0.005).

**Table 2:** Basic characteristics stratified by the median value of retinal age gap ^a^

Variables	Retinal age gap		Total (*n* = 566)	*P*-value
	Group 1 (*N* = 283)	Group 2 (*N* = 283)		
Screen exposure time (hours)				
Total	3.8 (1.84)	5.29 (2.2)	4.55 (2.16)	< 0.001
Light-on	3.74 (1.81)	5.18 (2.16)	4.46 (2.11)	< 0.001
Light-off	0.06 (0.25)	0.11 (0.33)	0.09 (0.3)	0.034
Retinal age gap (years)	− 1.21 (1.69)	6.71 (3.28)	2.75 (4.75)	< 0.001
Chronological age (years)	59.36 (1.63)	51.48 (3.46)	55.42 (4.78)	< 0.001
Outdoor duration (hours)	1.95 (1.29)	1.25 (1.08)	1.6 (1.24)	< 0.001
Sleep duration (hours)				
Total	7.93 (0.64)	7.72 (0.61)	7.82 (0.63)	< 0.001
Day	0.12 (0.32)	0.09 (0.3)	0.1 (0.31)	0.314
Night	7.81 (0.55)	7.63 (0.57)	7.72 (0.57)	< 0.001
Glucose (mmol/L)	6.15 (1.45)	5.9 (1.64)	6.03 (1.55)	0.052
BMI (kg/m^2^)	24.62 (3.01)	24.29 (3.32)	24.46 (3.17)	0.222
Hip circumference (cm)	82.63 (8.6)	80.52 (9.35)	81.57 (9.04)	0.005
Total cholesterol (mmol/L)	5.35 (0.99)	5.31 (1.02)	5.33 (1)	0.681
Mean arterial pressure (mmHg)	100.5 (10.16)	99.37 (12.25)	99.94 (11.26)	0.235
Sleep onset (time)	22.26 (0.66)	22.69 (0.61)	22.48 (0.67)	< 0.001
Gender No. (%)				0.859
Male	96 (33.92)	98 (34.63)	194 (34.28)	
Female	187 (66.08)	185 (65.37)	372 (65.72)	
Education No. (%)				< 0.001
Middle school and below	195 (68.9)	104 (36.75)	299 (52.83)	
High school and above	88 (31.1)	179 (63.25)	267 (47.17)	

^a^The participants were classified into two groups based on the median value of the retinal age gap: The participants’ retinal age gap was below the median value (< = 1.54 years) in Group 1, while those retinal age gap was higher than the median value in Group 2 (> 1.54 years). The continuous variables were in the form of mean (standard deviation) and were statistically tested by Student’s *t*-test; the categorical variables were in the form of No. (%), and were analyzed using chi-squared test. *BMI* body mass index

### Screen time exposure and retinal age gap

As shown in Table 1, the retinal age gap increased as the screen exposure time extended, from 0.49 ± 3.51 years in the lowest tertile to 5.13 ± 4.96 years in the highest tertile (ANOVA, *p* < 0.001; Jonckheere-Terpstra, *p* < 0.001). The group with larger retinal age gap also had significantly longer screen exposure (5.29 ± 2.2 h) compared with the group with smaller retinal age gap (3.8 ± 1.84 h, *p* < 0.001), no matter under bright environment or dark environment (Table 2).

Table 3 demonstrates the linear regression analyses for the relationship between retinal age gap and total screen exposure time. The screen exposure was linearly associated with the retinal age gap both in Model 1 and Model 2. For every 1 h increase in the total screen exposure time, the retinal age gap accelerated by 0.087 (95%CI, 0.027, 0.148, *p* = 0.005) years, adjusting for chronological age, gender, education, time outdoors, sleep duration, sleep onset time, fasting blood glucose, BMI, hip circumference, total cholesterol, and mean arterial pressure.

**Table 3:** Relationship between screen exposure time and retinal age gap

Variable	Model 1		Model 2	
	*β* (95% CI)	*P-*value	*β* (95% CI)	*P-*value
Screen exposure time	0.087 (0.028, 0.145)	0.004	0.087 (0.027, 0.148)	0.005

Model 1: adjusted for age, gender, and education

Model 2: adjusted for age, gender, education, outdoor duration, sleep duration, sleep onset, glucose, BMI, hip circumference, total cholesterol, and mean arterial pressure

*CI* confidence interval

### Mediation analyses based on the sleep traits

**Fig. 2:**
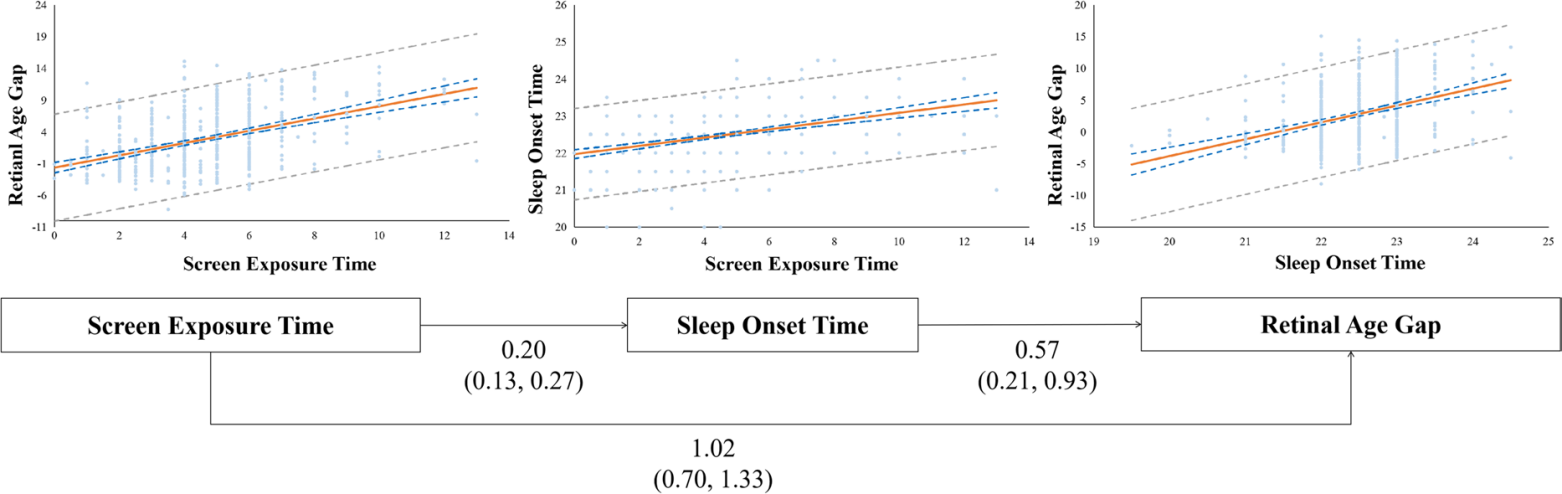
Relationship between screen exposure time, sleep onset time, and retinal age gap. This presents the scatter plots for screen exposure time and retinal age gap, screen exposure time and sleep onset time, and sleep onset time and retinal age gap. Below the scatter plots were the results of the mediation analyses. Longer screen time could increase the retinal age gap (*β* = 0.57, 95% CI 0.21 ~ 0.93) through the delayed sleep onset time (*β* = 0.20, 95% CI 0.13 ~ 0.27), and the proportions of indirect effect were 10.1%. Solid orange lines were the fitting lines for the linear regressions; blue dotted lines were the 95% confidence limits for the linear regressions; gray dotted lines were the 95% prediction limits for the linear regressions

Screen exposure time shortened sleep duration and accelerated aging (Table 4). However, sleep duration is not an intermediate variable between screen exposure time and retinal age gap. In the pathway analyses (Table 4), we observed that sleep onset time mediated the impact of screen usage on retinal age gap (indirect effect, *β* = 0.11; 95% CI 0.04 ~ 0.24), while sleep duration did not (indirect effect, *β* = 0; 95% CI − 0.03 to approximately 0.02). In detail, longer screen use may delay sleep onset time (*β* = 0.20, 95% CI 0.13 ~ 0.27), increasing the retinal age gap (*β* = 0.57, 95% CI 0.21 ~ 0.93) (Fig. 2). The proportion of indirect effect was 10.1%.

**Table 4:** Mediation effect of sleep duration and sleep onset time on the association between screen exposure and retinal age gap ^a^

Tested path		Model 1		Model 2	
		Mean (95% CI)	*P*-value	Mean (95% CI)	*P*-value
Screen usage—sleep duration—retinal age gap	**Total effect**	2.09 (1.75, 2.47)	< 0.001	1.02 (0.68, 1.33)	< 0.001
	**Direct effect**	2.03 (1.68, 2.40)	< 0.001	1.02 (0.68, 1.34)	< 0.001
	**Indirect effect**	0.06 (0.01, 0.16)	0.061	0 (-0.03, 0.02)	0.92
Screen usage—sleep onset time—retinal age gap	**Total effect**	2.09 (1.75, 2.43)	< 0.001	1.13 (0.82, 1.45)	< 0.001
	**Direct effect**	1.67 (1.31, 2.04)	< 0.001	1.02 (0.70, 1.33)	< 0.001
	Indirect effect^*^	0.42 (0.28, 0.59)	< 0.001	0.11 (0.04, 0.24)	< 0.01

^a^Mediation analysis was conducted using the Sobel test and Bootstrap method

Model 1: Unadjusted

Model 2: Adjusted for age, gender, education, outdoor duration, sleep duration, glucose, BMI, hip circumference, total cholesterol, and mean arterial pressure

^*^Model 1 mediation effect equals to 20.3% of total effect; Model 2 mediation effect equals to 10.1% of total effect

Further, we explored the impacts of screen usage in a light-on environment and in a light-off environment, respectively. We observed that sleep onset time mediated the impact of screen usage in a light-on environment (indirect effect, *β* = 0.42; 95% CI 0.27 ~ 0.57) on retinal age gap, and the proportion of indirect effect was 20.5%. In addition, sleep onset time fully mediated the impact of screen usage in a light-off environment (indirect effect, *β* = 0.22; 95% CI 0.07 ~ 0.38) on retinal age gap (Supplementary material 2-Table 1).

### Subgroup analyses for mediation analyses

Among the residents aged below 60-year-old, for every 1-h increase in the total screen exposure time, the retinal age gap accelerated by 0.088 (95% CI, 0.020, 0.156, *p* = 0.011) years; while among the residents aged over 60-year-old, the impact of the total screen exposure time on the retinal age gap was not significant (*p* = 0.081) (Supplementary material 2-Table 2).

In addition, the pathway analysis confirmed the results above. Among the residents aged below 60 years old, sleep onset time mediated the impact of screen usage on retinal age gap, and the proportion of indirect effect was 23.7%. However, among the residents aged over 60 years old, the increase in the total screen exposure time did not delay the residents’ sleep onset time (Supplementary material 2-Table 3).

## Discussion

Our study found that in the middle-aged and elderly population, excessive time spent in screen devices was associated with larger retinal age gap, which might be mediated by the delayed sleep onset time. This phenomenon was pronounced in residents younger than 60 years of age and might not be applicable to residents over 60 years of age. Although screen exposure time shortened sleep duration and accelerated aging, sleep duration is not an intermediate variable between screen exposure time and retinal age gap.

To the best of our knowledge, there is no study on excessive screen exposure and accelerated retinal aging. Normal use of LED light devices is not deleterious to human retina [32]. However, the potential toxicity of long-term cumulative exposure to the LED light devices is not determined in human eyes due to the difficulty to accurately measure the exposure [33]. Blue light which was enriched in LED devices could induce and accelerate cellular damage and photochemical reactions through the generation of ROS, resulting in lipid peroxidation, the loss of photoreceptors, and retinal pigment epithelium (RPE) cell dysfunction or apoptosis [34, 35]. These processes are similar to those observed in age-related macular degeneration (AMD), a senile disease with increasing prevalence with age, in which the photoreceptors degenerate while the RPE cells accumulate phototoxic components [36]. These were also coincided with the aging retina where the RPE cells were manifested by the accumulation of lipofuscin and formation of drusen, and the number of photoreceptor cells were decreased [37]. Moreover, with age, the antioxidant defenses are weakened making them incapable of neutralizing the oxidative stress produced by excessive exposure to blue light, which, in turn, could also expedite the damage of blue light to the human retina [38]. Therefore, theoretically, long-term exposure to screen could lead to retinal aging.

Study on screen exposure and biological aging is also scarce. The retinal age gap represents the accelerated process of aging and is related to a number of senile diseases [24, 26–29]. The present study found that prolonged screen exposure could speed up aging through the delay of sleep onset, which could be an indicator of delayed circadian clock. Existing literatures have suggested that excessive screen exposure, especially at night, could lead to changes in circadian rhythm, such as delayed sleep onset, reduced next-morning alertness, and decreased melatonin secretion [6, 10, 12, 13]. It has also been proven that blue light, which is abundant in screen devices, can disrupt the circadian rhythm [32, 39]. Meanwhile, changes in circadian rhythms could lead to the acceleration of aging and the shortened lifespan both in animals and human beings [17–19, 40–42]. In addition, in the process of aging, the damage of the retina caused by the blue light could lead to a decline in neuronal excitability and synaptic transmission [39]. It was reported that excessive screen time could alter gray matter and white volumes in the brain, undermine acquisition of memories and learning, and increase the risk of mental disorders and dementia [43]. All these evidence support the findings in the present study.

According to mediation analyses, it was indicated that sleep duration did not serve as a mediator between screen exposure and the retinal age gap; however, sleep onset time did. One possible explanation for the absence of a correlation in the mediation effect is that the relationship between sleep duration and aging could be nonlinear. In other words, both excessive and insufficient sleep duration may lead to accelerated aging [16, 44, 45], thus negating a mediating role. Conversely, a delayed sleep onset suggests a lag in the release of melatonin and disturbances in the circadian rhythm, which are closely associated with the acceleration of aging [17–19, 40–42]. Further analyses showed that the impact of screen usage in the light-off environment on the retinal age gap was fully mediated by sleep onset time, while the proportion of indirect effect of sleep onset time in the light-on environment was only 20.5% (Supplementary material 2-Table 1). This also suggested that circadian rhythm disruption may mediate the relationship between screen exposure and aging.

The phenomenon was observed in subgroup of residents younger than 60 years old, but not in the residents older than 60 years. Given that 60 is the retirement age in our country, individuals below this age are typically engaged in work. Consequently, their screen time tends to be more concentrated in the evenings, after work, which subsequently increases the likelihood of delayed sleep onset. Conversely, those who have reached or exceeded the age of 60 years, being rehired after retirement and often engaging in some leisure jobs, could have adequate time to use screens during the day, resulting in a lower likelihood of experiencing delayed sleep onset among this elderly population. Furthermore, as one ages, the crystalline lens of the eye becomes increasingly dense, thereby allowing less blue light to penetrate into the retina [33, 46]. Consequently, this reduced exposure to blue light decreases the likelihood of disrupting the circadian rhythm, ultimately contributing to the aging process.

The strength of this study lies in its comprehensive design, encompassing general and ophthalmological examinations, as well as questionnaires conducted among the community population. Rigorous statistical analyses were employed to arrive at the present conclusions. However, there are limitations to be noted. Firstly, the study is cross-sectional, lacking longitudinal follow-up, thus preventing the establishment of a causal relationship, and merely suggesting a correlation. Secondly, aging was assessed through the calculation of the retinal age gap from fundus photographs, lacking additional indicators for a more comprehensive evaluation of aging. Thirdly, sleep time, sleep onset time, and screen exposure time were all obtained via questionnaires, introducing the potential for recall bias. Future studies should aim to incorporate more objective measures for these variables, incorporate additional aging indicators for validation, and include longitudinal follow-up to establish causal relationships.

## Conclusions

Our study revealed that, among middle-aged and elderly individuals, excessive screen time was linked to a wider retinal age gap. This may be partly explained by delayed sleep onset time. Notably, this trend was more evident among those below 60 years of age and may not extend to those over 60. Therefore, for working population, it is necessary to control screen time and avoid using screens before bedtime to reduce the impact of screen on the retina and systemic aging.

## Supplementary Information

Below is the link to the electronic supplementary material.

Supplementary file1 (DOCX 13 KB)

Supplementary file2 (DOCX 16 KB)

## Data Availability

The data that support the findings of this study are not openly available due to reasons of sensitivity and are available from the corresponding author upon reasonable request. Data are in controlled access data storage at Shanghai Eye Disease Prevention and Treatment Center.

## Abbreviations

LED: Light-emitting diodes
DL: Deep learning
RPE: Retinal pigment epithelium
AMD: Age-related macular degeneration
