# Supplementary material for: Sleep onset time as a mediator in the association between screen exposure and aging: a cross-sectional study
Source: GeroScience. 2024 Aug 27;47(1):1239–49. doi: 10.1007/s11357-024-01321-x (PMC11872958; doi:10.1007/s11357-024-01321-x)
Supplement: Supplementary file 1 — Supplementary file1 (DOCX 13 KB) [file 11357_2024_1321_MOESM1_ESM.docx]

**Construction process of the AI model based on color fundus images for age prediction**

A total of 917 color fundus images were used for the development of the AI model, with an average age of 66.45, a standard deviation of 11.33, a maximum age of 92, and a minimum age of 22. Ninety percent of these images were used as the training set, while the remaining 10% were employed as an internal validation set.

All images in the training dataset were resized to 256×256 pixels and underwent data augmentation, including random cropping, random horizontal flipping, and image normalization. After preprocessing, images were fed into our age-predicting model using the ResNet50 architecture. The model was trained on our dataset with the aim of predicting the age of the patients corresponding to the fundus images. Training was conducted using 2 NVIDIA GeForce RTX 2080 Ti GPUs, with a batch size of 16, over 50 epochs. To improve training outcomes and reduce overfitting, a cosine annealing method was used to update the learning rate: it gradually increased from 0 to 5×10^-4 over the first 10 epochs and then progressively decreased from 5×10^-4 to 1×10^-6 over the remaining 40 epochs. After each training epoch, the model was evaluated on the internal validation dataset, and the model weights with the lowest mean absolute error (MAE) were saved. The correlation between predicted retinal age generated by the trained DL model and chronological age was 0.76 (p<0.001), with an overall MAE of 5.83 years.
