# Supplementary material for: Sleep onset time as a mediator in the association between screen exposure and aging: a cross-sectional study
Source: GeroScience. 2024 Aug 27;47(1):1239–49. doi: 10.1007/s11357-024-01321-x (PMC11872958; doi:10.1007/s11357-024-01321-x)
Supplement: Supplementary file 2 — Supplementary file2 (DOCX 16 KB) [file 11357_2024_1321_MOESM2_ESM.docx]

**Table S1. Mediation effect of sleep onset time on screen exposure time (stratified by exposure time under bright or dark environment) and retinal age gap**

|  | **Screen Exposure under Light-on Environment ^a^** | | **Screen Exposure under Light-off Environment ^b^** | |
| --- | --- | --- | --- | --- |
|  | **β (95%CI)** | ***P* value** | **β (95%CI)** | ***P* value** |
| Total Effect | 2.06 (1.73, 2.42) | <0.001 | 0.54 (0.12, 0.74) | 0.006 |
| Direct Effect | 1.64 (1.30, 2.00) | <0.001 | 0.32 (-0.04, 0.72) | 0.081 |
| Indirect Effect | 0.42 (0.27, 0.57) | <0.001 | 0.22 (0.07, 0.38) | <0.01 |

1. The mediation effect of delayed sleep onset time on aging caused by screen exposure in light-on environments accounts for 20.5% of the total effect of aging caused by screen exposure in light-on environments. This result suggests that there may be other ways through which screen exposure time under light-on environment affects aging.

b. After excluding the mediation effect of delayed sleep onset time on aging caused by screen exposure in light-off environment, the direct effect is no longer significant, indicating that delayed sleep onset is the primary pathway through which screen exposure in light-off environments affects aging.

**Table S2. Subgroup analyses for relationship between screen exposure time and retinal age gap by chronological age**

| **Variable** | **Age <60** | | **Age ≥60** | |
| --- | --- | --- | --- | --- |
|  | ***β* (95%CI)** | ***P* value** | ***β* (95%CI)** | ***P* value** |
| **Screen Exposure Time** | 0.088 (0.020, 0.156) | 0.011 | 0.127 (-0.016, 0.269) | 0.081 |

Adjusted for age, gender, education, outdoor duration, sleep duration, sleep onset, glucose, BMI, hip circumference, total cholesterol, and mean arterial pressure.

CI = confidence interval

**Table S3. Subgroup analyses for mediation effect of sleep onset time on the association between screen exposure time and retinal age gap by chronological age ^a^**

|  | **Impact of screen exposure time on sleep onset**  **β (95%CI)** | ***P* value** | **Impact of sleep onset on retinal age gap**  **β (95%CI)** | ***P* value** | **Proportion** |
| --- | --- | --- | --- | --- | --- |
| **Age <60*** | 0.36 (0.27, 0.44) | <0.001 | 1.16 (0.78, 1.53) | <0.001 | 23.7% |
| **Age ≥60** | 0.19 (-0.02, 0.41) | 0.07 | / | / | / |

*: Sobel test p<0.001; unadjusted. The mediation effect accounts for 23.7% of the total effect.

Increased screen exposure time delayed sleep onset time and lead to accelerated aging in individuals under 60 years old. However, in individuals aged 60 and older, increased screen exposure time did not delay sleep onset time, and therefore not leading to accelerated aging.
